# A kNGR Peptide-Tethered Lipid–Polymer Hybrid Nanocarrier-Based Synergistic Approach for Effective Tumor Therapy: Development, Characterization, Ex-Vivo, and In-Vivo Assessment

**DOI:** 10.3390/pharmaceutics14071401

**Published:** 2022-07-03

**Authors:** Madhu Gupta, Vikas Sharma, Kalicharan Sharma, Anoop Kumar, Ajay Sharma, Imran Kazmi, Fahad A. Al-Abbasi, Sami I. Alzarea, Obaid Afzal, Abdulmalik Saleh Alfawaz Altamimi, Sachin Kumar Singh, Gaurav Gupta, Keshav Raj Paudel, Philip M. Hansbro, Kamal Dua

**Affiliations:** 1 Department of Pharmaceutics, Delhi Pharmaceutical Science and Research University, Pushp Vihar Sector-3, MB Road, New Delhi 110017, India; madhu@dpsru.edu.in (M.G.); kcsharma@dpsru.edu.in (K.S.); anoopniper@gmail.com (A.K.); 2 Drug Delivery Research Laboratory, Department of Pharmaceutical Sciences, Dr. H.S. Gour University (A Central University), Sagar 470003, India; 3 Divine International Group of Institutions-Pharmacy, Gwalior 474001, India; vikassharma15@gmail.com; 4 Department of Biochemistry, Faculty of Science, King Abdulaziz University, Jeddah 21589, Saudi Arabia; ikazmi@kau.edu.sa (I.K.); fabbasi@kau.edu.sa (F.A.A.-A.); 5 Department of Pharmacology, College of Pharmacy, Jouf University, Sakaka 72388, Saudi Arabia; samisz@ju.edu.sa; 6 Department of Pharmaceutical Chemistry, College of Pharmacy, Prince Sattam Bin Abdulaziz University, Al Kharj 11942, Saudi Arabia; o.akram@psau.edu.sa (O.A.); as.altamimi@psau.edu.sa (A.S.A.A.); 7 School of Pharmaceutical Sciences, Lovely Professional University, Phagwara 144411, India; sachin.16030@lpu.co.in; 8 Faculty of Health, Australian Research Centre in Complementary and Integrative Medicine, University of Technology Sydney, Ultimo 2007, Australia; 9 School of Pharmacy, Suresh Gyan Vihar University, Mahal Road, Jaipur 302017, India; drgaurav.gupta@mygyanvihar.com; 10 Department of Pharmacology, Saveetha Dental College, Saveetha Institute of Medical and Technical Sciences, Saveetha University, Chennai 602105, India; 11 Uttaranchal Institute of Pharmaceutical Sciences, Uttaranchal University, Dehradun 248007, India; 12 Centre for Inflammation, Centenary Institute and University of Technology Sydney, Faculty of Science, School of Life Sciences, Sydney 2007, Australia; keshavraj.paudel@uts.edu.au; 13 Discipline of Pharmacy, Graduate School of Health, University of Technology Sydney, Sydney 2007, Australia

**Keywords:** kNGR peptide, hybrid nanoparticles, targeted therapy, intracellular delivery, polymer-lipid CD13 receptor

## Abstract

The present study aims to design, develop and characterize kNGR (Asn-Gly-Arg) peptide-conjugated lipid–polymer-based nanoparticles for the target-specific delivery of anticancer bioactive(s), i.e., Paclitaxel (PTX). The kNGR-PEG-DSPE conjugate was synthesized and characterized by using spectral analysis. The dual-targeted PLGA–lecithin–PEG core-shell nanoparticles (PLNs-kNGR-NPs) were synthesized using a modified nanoprecipitation process, and their physiological properties were determined. The results support that, compared to other NPs, PLNs-kNGR-NPs are highly cytotoxic, owing to higher apoptosis and intracellular uptake. The significance of rational nanoparticle design for synergistic treatment is shown by the higher tumor volume inhibition percentage rate (59.7%), compared to other designed formulations in Balb/c mice in the HT-1080 tumor-induced model. The overall results indicate that the PLNs-kNGR-NPs-based hybrid lipid–polymer nanoparticles present the highest therapeutic efficacy against solid tumor overexpressing the CD13 receptors.

## 1. Introduction

Cancer is one of the leading causes of death throughout the globe. Scientists are exploring new treatment possibilities for the successful management of cancer. Chemotherapy is one of the important regimens for cancer therapy [1]. The limited success of conventional monotherapy concurrent with toxicity and drug resistance led to the development of combination therapy. Nevertheless, commercially available cytotoxic agents possess several side effects and create toxicity due to non-specificity towards normal and cancerous cells [2]. The targeted delivery system(s) has been explored specifically to target the tumor cells, hence overcoming the side effects of conventional chemotherapy. These systems mainly consist of targeting ligand, a carrier system, and bioactive moiety [3]. Many nanomodules and newer fascinating devices, namely: liposomes, polymeric nanoparticles, dendrimers or lipidic nanoparticles, polymer–lipid hybrid nanoparticles (PLNs), micelles, carbon nanotubes, and nanotubes have been crafted for enhancing the transport of therapeutic cargo to treat the tumor cells [4,5,6]. These nanoparticles have been used for drug administration by combining the advantages of polymeric core particles, liposomes, and polymer–lipid–PEG hybrids [7]. They show significant benefits, such as higher drug loading, and can incorporate both hydrophilic and hydrophobic molecules [8,9,10,11,12]. These nanosystems consist of a hydrophobic PLGA core, a lipid monolayer shell (lecithin) around the polymer core, and a PEG-linked lipid interposed in the lecithin layer, which forms the outer of the nanocarrier hydrophilic [13,14]. The lipid monolayer offers stability issues and outward diffusion of the encapsulated drug, providing support for attachment of specific diseased ligands, which aids in the targeted delivery of bioactives compounds [15,16]. The characteristics of nanoparticles may be altered by changing the material composition to customize them to specific medicinal needs. The PLNs emerged as a promising carrier system due to their excellent stability and targeting abilities, as many ligands can be attached [17,18]. Numerous targeting alternatives have been discovered that selectively detect and bind to the tumor cell receptors that are overexpressed [19,20]. CD13 is a multifunctional protein receptor involved in tumor angiogenesis, invasion, and metastasis [21,22]. NGR-based peptides have been used to deliver the cancer drug DOX, apoptotic peptides, and cytokines such as the tumor necrosis factor for transporting the tumor or tumor vasculature and augmenting the therapeutic efficacy [23,24]. The NGR (Asn-Gly-Arg) is a short peptide that can recognize and bind with a specific isoform of CD13 receptors and has a high-affinity interaction with the NGR peptide [25,26]. The broad usage of NGR peptide sequences as ligands for NGR-targeted drug and gene delivery applications has been attributed to CD13 proteins.

**Figure 1:**
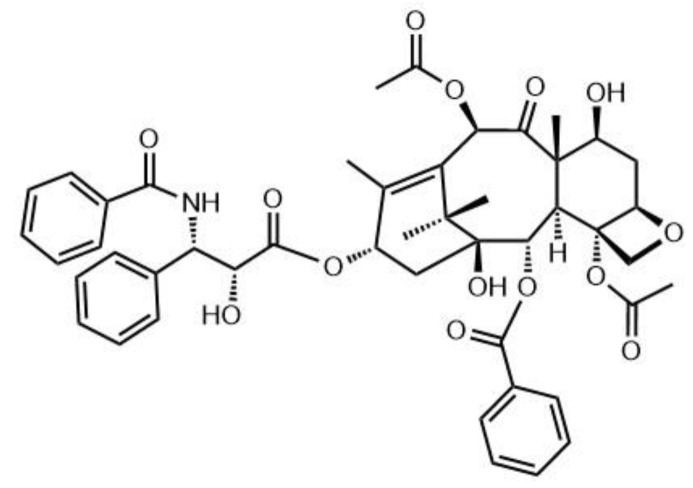
Chemical structure of paclitaxel/taxol.

Paclitaxel (PTX)/Taxol (Figure 1) is an anticancer drug widely used for its significant anti-cancerous activity, including melanoma, non-small cell lung carcinoma, head and neck cancer, ovarian and breast cancer, and AIDS-related cancer [27]. However, PTX has a shorter circulation half-life, low water solubility, and drug resistance, leading to side effects. However, its clinical potential is compromised due to its side effects and nonspecific transport in-vivo when delivered in conventional formulations [28]. The purpose of this research is to provide proof-of-concept for the hypothesis that kNGR-modified PLGA-Lecithin-PEG (PLNs-kNGR) nanoparticles containing paclitaxel can attach precisely to the tumor endothelial cells and inhibit the proliferation of tumor cells. Here, HUVEC (Human umbilical vein endothelial cell) and HT-1080 (Human fibrosarcoma cell line) were selected to represent the blood endothelial cells and solid tumor cells, and used for further studies. Furthermore, the in-vitro targeting properties of NGR-modified NPs were investigated by membrane integrity assay, clonogenic assay, and cellular apoptosis. In addition, the in-vivo antitumor abilities, plasma distribution study, and other toxicological parameters were carried out on tumor-induced Balb/c mice bearing the HT-1080 tumor model.

## 2. Materials and Methods

### 2.1. Materials

Paclitaxel (PTX) and Poly-lactide/glycolide (PLGA) were gifted by the Sun Pharma advanced research lab, Vadodara, India. kNGR peptide was synthesized and purchased from USV Ltd. Mumbai, India. 1,2 distearoyl-sn-glycero-3-phosphoethanolamine-N-[carboxy (polyethylene glycol)-200] was procured from Avanti, USA. Fluorescein isothiocyanate (FITC), DNAse-free RNAse and 3-(4,5-dimethyl thiazol-2-yl)-2,5-diphenyltetrazolium bromide (MTT) and Propidium iodide (P.I.) were procured from Sigma Aldrich, St. Louis, MO, USA.

### 2.2. Synthesis and Characterization of DSPE–PEG–kNGR Conjugate

**Figure 2:**
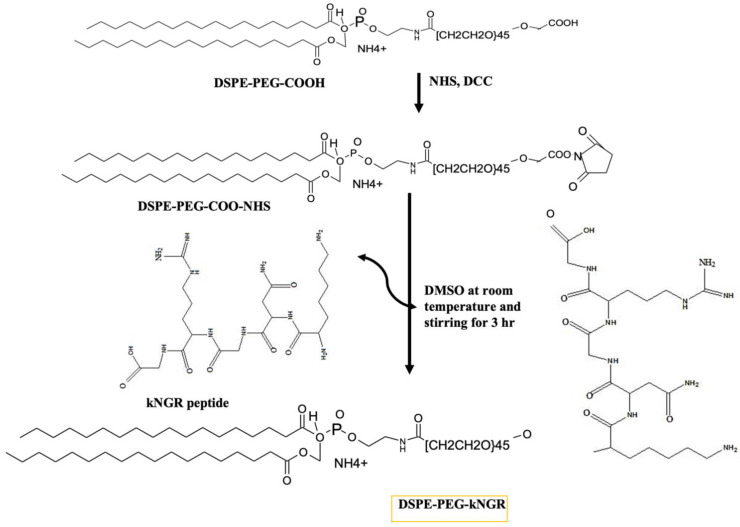
Graphical scheme of synthesis of the DSPE-PEG-kNGR conjugate.

DSPE–PEG–kNGR conjugate was synthesized using kNGR peptide and DSPE-PEG_2000_-COOH with the previously reported method (Figure 2) [9]. FT-IR was used to characterize the synthesized DSPE–PEG–kNGR (Thermo Nicolet Nexus 670, Madison, WI, USA).

### 2.3. Preparation and Physicochemical Characterization of Polymer–Lipid Hybrid N.P.s (PLNs)

PTX loaded PLNs were prepared by a modified nanoprecipitation technique as reported previously in the literature. Polymer–lipid hybrid N.P.s (PLNs) were designed and structured from Lecithin (P.C.), PLGA, and DSPE-PEG_2000_-COOH. Optimized polymer–lipid hybrid N.P.s were characterized for shape and surface morphology by high-resolution transmission electron microscopy (Leo 435 VP501B, Philips) [29]. Photon correlation spectroscopy (PCS) was used to assess particle size, zeta potential, and PDI using a Malvern Zeta sizer at 25 °C and 120 s equilibrium time. The % entrapment efficiency was determined with the HPLC system (Shimadzu, Japan) following centrifugation (25 × 10^3^ rpm for 30 min) [30]. The efficiency and density of kNGR on the PLNs’ surface were accessed using the CBQCA Protein Quantitation Kit [31]. The release of PTX from LPNs was determined as reported in previous studies in phosphate (pH 7.4) and phthalate buffer (pH 5.0). The adsorption of protein on the surface of PLNs and hemolytic toxicological studies were also performed by the previously reported methods (Supplementary Materials).

### 2.4. Cell Culture

The current work used the human fibrosarcoma cell line (HT-1080) and human umbilical vein endothelial cells (HUVEC cells) obtained from the National Centre for Cell Science in Pune, India. In a 5 percent CO_2_ incubator, cells were incubated in a new DMEM medium with 10% FBS. First, the culture flask was removed from the culture cab without causing any disruption to the media, and the surface was wiped with 90% alcohol. Next, the medium was removed, and TPVG solution was used to rinse it (0.2 percent trypsin, 0.02 percent EDTA, 0.05 percent glucose in PBS). Finally, the solution was withdrawn, 1ml of TPVG solution was added, and the flask was held at 37 °C until the cells were detached, after which fresh medium supplements (pH 7–8) were added, aspirated, distributed into new flasks, and transferred to the CO_2_ incubator at 37 °C and 5% CO_2_.

### 2.5. Trypan Blue Exclusion Assay and Clonogenic Assay

The anticancer activity of nanoparticulate systems was tested using the previously disclosed Trypan blue exclusion assay and the previously reported technique [32,33] (Supplementary Materials).

### 2.6. Cell Apoptosis and Cytotoxicity Assay

The cell cytotoxicity tests were carried out on both HT-1080 and HUVEC cells. 5 × 10^3^ cells per well were sown in a 96 well flat-bottom plate and allowed to mature for 24 h. The apoptosis activity of the cells was assessed by seeding the cells in 96 well flat-bottom plates containing a coverslip with 2 × 10^5^ cells /well at 37 °C for 24 h [34].

### 2.7. Cell Cycle Analysis

A cell cycle study was performed on HT-1080 cells, seeded at a density of 5 × 10^6^ cells per well in a 6-well plate and allowed to grow for 24 h, incubated with 1 mL media containing formulations further incubated for 36 h. The cells were washed in PBS and treated with trypsin-EDTA (200 µL) before being collected and centrifuged for 5 min at 1500 rpm after the media was removed. Cells were resuspended in cold PBS, vortexed, and 2 mL absolute ethanol was added drop-by-drop to achieve a final concentration of 70% *v/v*. Cells were incubated for 15 min at 4 °C, and then resuspended in 250 μL staining PBS solution (RNAse A (0.1 mg/mL), Triton-X 100 (0.05% *v/v*) and PI (10 μg/mL). After incubation for 1hr at R.T. in the dark, cells were analyzed with BD FACS (Bioscience, USA) after 1 h incubation at room temperature. Data were recorded and analyzed using Cell quest software.

### 2.8. Cellular Uptake of PLNs and Competition Assay

The cellular uptake assessment and competition assay were performed using HT-1080 cells as per the reported procedure.

### 2.9. In-Vivo Studies

The Institutional animal ethical committee of Dr. Harisingh Gour University, Sagar (M.P.), authorized the in-vivo experimental procedure for animal experiments through letter no. Animal Eths. Comm.11/10/87, and the investigations were carried out according to the protocol approved by the CPCSEA committee.

### 2.10. In-Vivo Antitumor Activity

Balb/c mice (6–8 weeks, 20–25 gm) harboring HT-1080 cells were used to test the antitumor effectiveness. 2 × 10^6^ cells were injected S.C. in the flank of mice. The day when the tumor volume reached about 100 mm^3^ was designated as day 0 (Supplementary Materials). Mice were randomly divided into groups as per approved protocol (*n* = 9) and treated with one of the dosing regimens. The animals of each group were treated with a 4-day gap with each respective formulation by tail vein injection. Tumor growth and weight loss were monitored and measured, and survival time was assessed. In addition, the percentage regression of tumor volume (P.I.) and percentage tumor weight inhibition (W.I.) were calculated [35].

### 2.11. Biodistribution Studies and In-Vivo Toxicological Parameters

The biodistribution of PTX-loaded formulations in plasma and tissue was performed on tumor-induced Balb/c mice. In addition, the various toxicological parameters, such as hematological, nephrotoxic, and hepatotoxic effects, were also performed on Balb/c mice (Supplementary Materials).

### 2.12. Data Analysis

All the results are expressed as mean ± standard deviation. The treated groups were compared to the control by analysis of variance (ANOVA), following Dunnet’s test. The statistical analysis was carried out using Instat 2.1 software, Graph Pad Software Corp., San Diego, CA, USA. The *p*-value < 0.05 was considered significant.

## 3. Results

### 3.1. Synthesis and Characterization of DSPE–PEG–kNGR Conjugate

To enhance the specificity and targeting efficiency against the CD13 receptor over-expressed on HT-1080 and HUVEC cells, the kNGR peptide was conjugated to the carboxyl group of DSPE-PEG-COOH through the NHS and DCC method. The detailed analysis was obtained on a supplementary file (Figure S2).

Analysis of the product by FT-IR showed important peaks for DSPE–PEG–kNGR at 1745 cm^−1^ (ester conjugated C=O), 2851 and 2929 cm^−1^ (aromatic C=C bending and stretching), 807.5 cm^−1^ (aromatic C-H bending), 3327 cm^−1^ (N-H stretch of primary amine and amide), and 1418 cm^−1^ (C-N stretching) (Figure S1).

### 3.2. Physicochemical Characterization of PLNs

**Figure 3:**
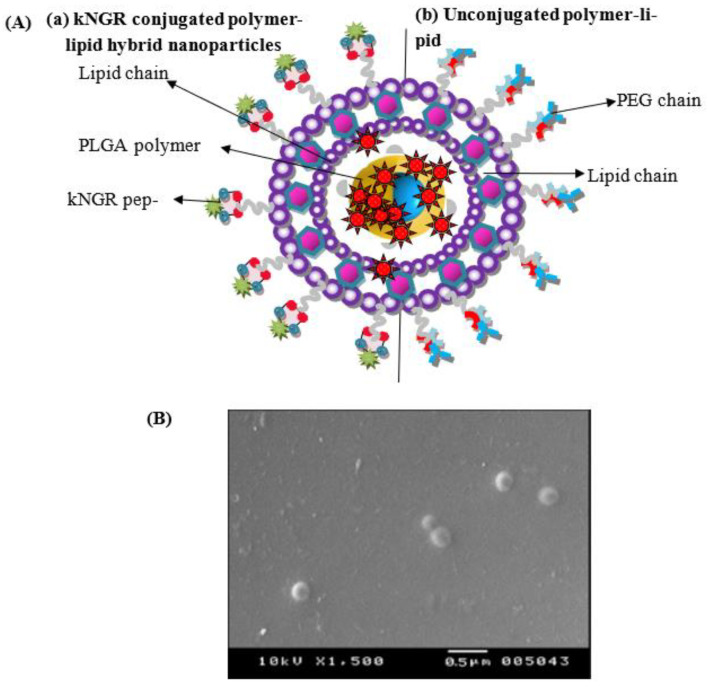
(**A**) Structure of polymer–lipid hybrid nanoparticles with schematic comparison of (**a**) kNGR-conjugated polymer–lipid hybrid nanoparticles (**b**) unconjugated polymer–lipid hybrid nanoparticles (**B**) SEM photograph of PLNs-kNGR.

The modified nano-precipitation technique was used to fabricate self-assembled PLNs with an inner polymeric PLGA core (hydrophobic), stealth shell of DSPE–PEG (hydrophilic polymer), and lecithin monolayer at the interface of core and shell (Figure 3A). The aqueous phase self-assembled on the surface of the hydrophobic PLGA core consisted of P.C. and PEGylated phospholipids (DSPE–PEG_2000_) in a suitable molar ratio. It can provide the aqueous solubility, composite integrity, and diffusion barrier for encapsulated drugs. The lipid aided the thermal and mechanical energy and scattered as a layer around the PLGA core [36]. The hydrophilicity of PLNs restricts the RES uptake and accumulation of the PLNs, acting as a linker for surface conjugation. The morphology of PLNs-kNGR was observed using SEM, which showed a smooth, spherical shape, nanometric in size range (Figure 3B). Table 1 summarizes the physicochemical features of the PLNs. The mean diameter of ligand-conjugated PLNs had a larger size, 205 nm, with a higher drug % EE 82.21% compared to unconjugated PLNs. In addition, the ligand-conjugated PLNs demonstrated slightly more negative zeta potential.

As indicated in Table 1, the effectiveness of kNGR peptide conjugation was 34.7 percent, while the surface density on the PLNs’ surface was 198. This is due to the multivalent array of kNGR peptidic ligand on the surface of N.P.s, which allows for better binding to CD13 receptors. As shown in Figure S2, the PLNs and PLNs-kNGR exhibited an initial fast release, followed by sustained drug release from PLNs at pH 7.4 and pH 5.0. About 27.7% PTX from targeted kNGR-oriented PLNs was released in PBS at pH 7.4 (Figure S2a), while 46.5% of the drug was removed from PLNs-kNGR at pH 5.0, respectively, in 24 h (Figure S2b).

The plasma protein adsorption studies suggest that the particle size of PLNs and PLNs-kNGR remained nearly unchanged after 24 h incubation with either 5% FBS or 2% BSA media, due to steric prevention of outer PEG; the adsorption of protein of the plasma is not permitted, hence clumping could be prevented (Figure S3). The hemolytic study suggests that in the case of PLNs-NPs and ligand-conjugated N.P.s (PLNs-kNGR), considerably decreased hemolysis is observed at all concentrations (Figure S4). The presence of the PEG layer and ligand moiety on the PLNs surface accounts for the lower hemolytic activity.

### 3.3. Cell Membrane Integrity and Anticancer Activity

Figure S5 presents the normalized membrane integrity in the case of PLNs-kNGR-NPs on prolonged incubation. Among the PTX formulations, PLNs-kNGR-NPs showed a higher diminishing effect on membrane integrity. When administered in the concentration range of 2.5 µg/mL in HT-1080 cells, the PLNs-kNGR produced much greater hazardous and cell-eradicating outcomes. While in the case of HUVEC cells the PLNs-kNGR-based treatment presented lower PE 1.54 and 1.34-fold than PTX-NPs and PLNs-based treatments (Figure S6).

### 3.4. Evaluation of Cell Apoptosis Activity

**Figure 4:**
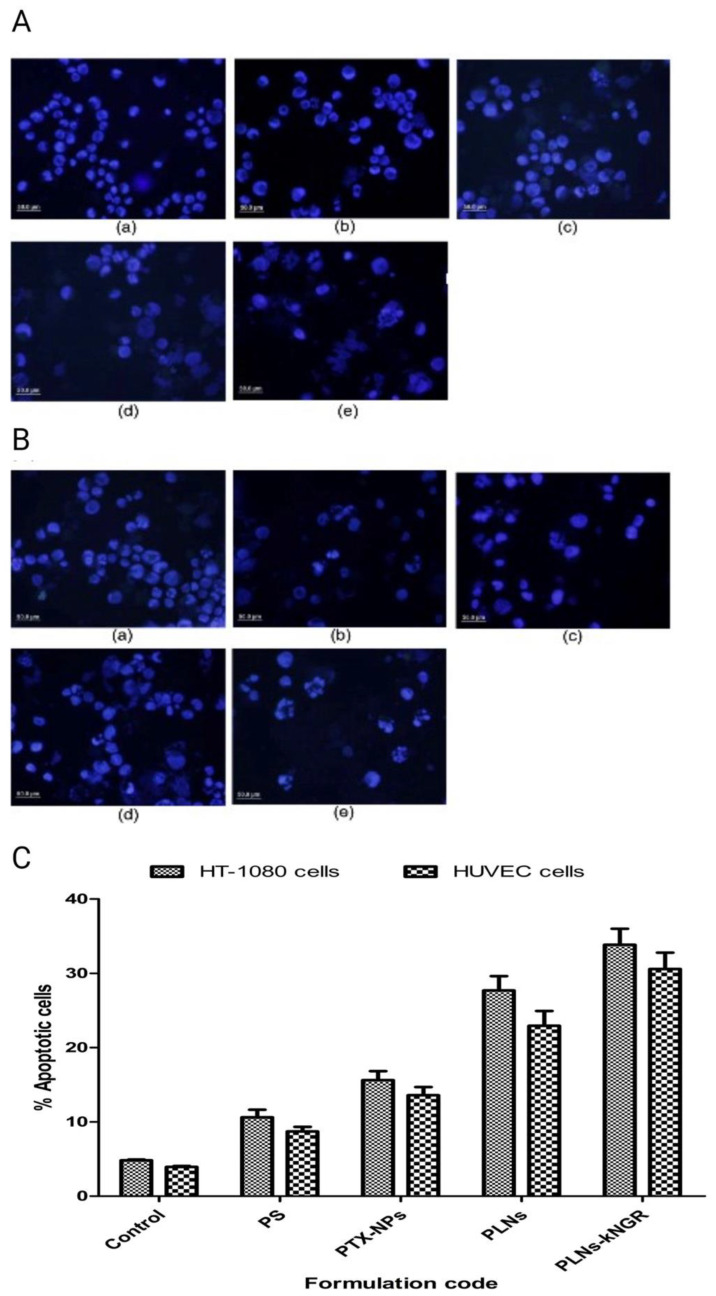
Influence of PTX and PTX-loaded NPs on nuclear morphology and apoptotic bodies formation in (**A**) HT-1080 cells and (**B**) HUVEC cells; (**a**) Untreated control cells (**b**) Cells treated with free PTX solution (PS) (**c**) PTX-NPs (**d**) PLNs (**e**) PLNs-kNGR. (**C**) Percent of apoptotic cells in HT-1080 and HUVEC cell line after 36 h treated with various formulations in the section. (Bar 50 µm).

The apoptotic morphology of HT-1080 and HUVEC cells was studied after different formulations were treated. The results suggest that the percentage of the apoptotic cells was much higher in the case of kNGR peptide-conjugated PLNs for both types of cells (Figure 4).

### 3.5. Cytotoxicity Assay

**Figure 5:**
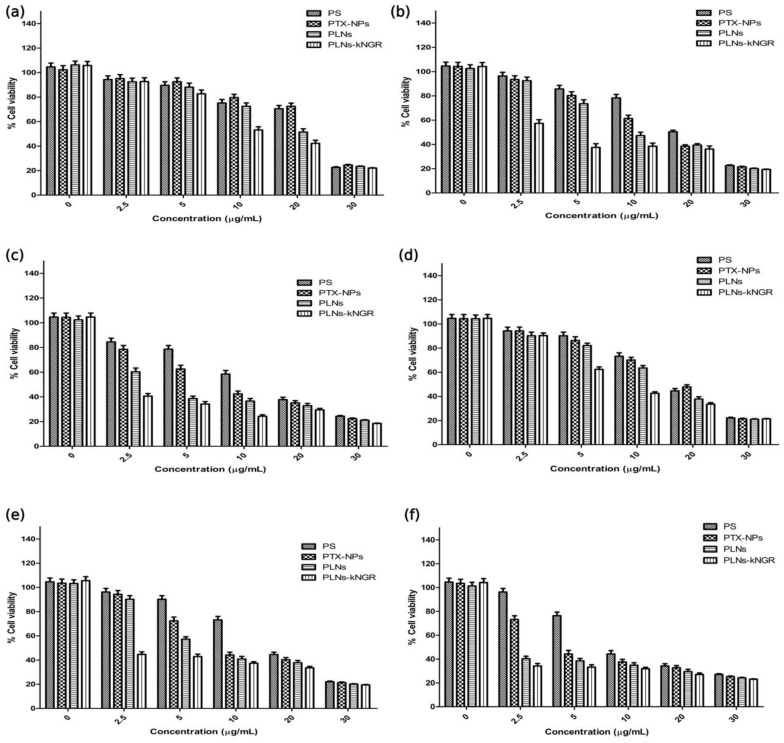
In-Vitro cytotoxicity at different concentration of various PTX-based formulations as free PTX solution (PS) PTX-based Nanoparticles (PTX-NPs), unconjugated polymer–lipid hybrid nanoparticles (PLNs) and kNGR-conjugated polymer–lipid hybrid nanoparticles (PLNs-kNGR) of PTX against different cells (**a**) 24 h (**b**) 48 h (**c**) 72 h and in HT-1080 cells (**d**) 24 h (**e**) 48 h (**f**) 72 h in HUVEC cells.

PLNs-kNGR exhibited the maximum inhibitory effect on the proliferation of HT-1080 and HUVEC cells among various formulations at all concentrations (Figure 5). The anti-proliferation ability of the different formulations followed the order: PLNs-kNGR > PLNs > PTX-NPs > P.S (Table 2).

### 3.6. Cell Cycle Analysis

**Figure 6:**
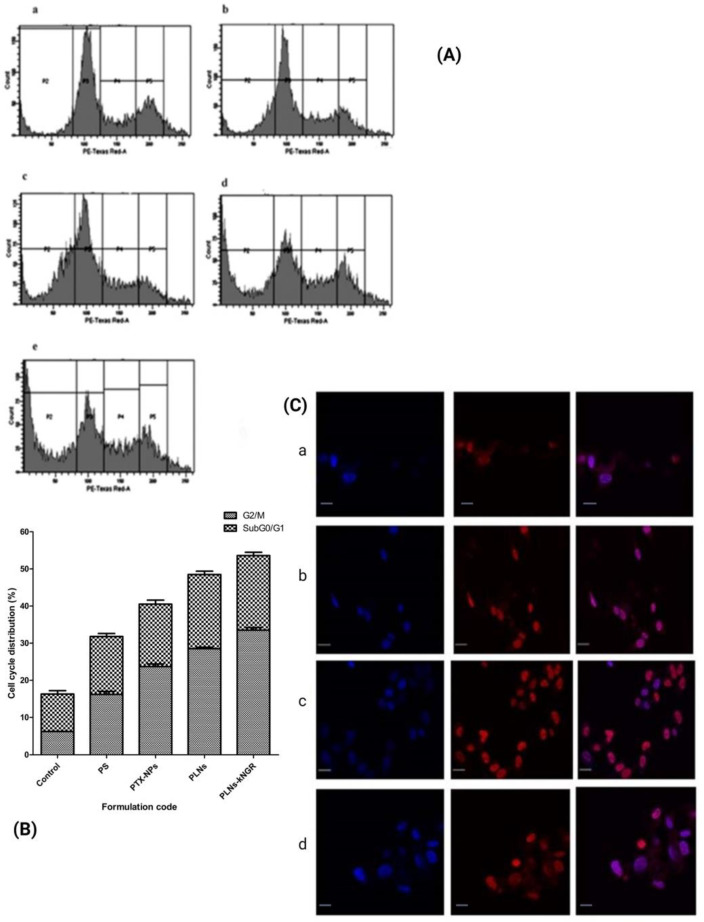
(**A**) Representative photomicrographs of cell cycle distribution in HT-1080 cells. Cells were incubated in the presence of various PTX-loaded formulations at equivalent drug concentration of 0.5 µg/mL for 36 h and analyzed by flow cytometry. Areas P2, P3, P4 and P5, represent sub-G0/G1, G0/G1, S and G2/M phases, respectively, of the cell cycle. (**A**) (**a**) Control (**b**) PS (**c**) PTX-NPs (**d**) PLNs (**e**) PLNs-KNGR. (**B**) Kinetics of distribution of the G2/M and sub-Gs0/G1 population induced by PTX-based formulations. (**C**) Cellular association of various formulations in HT-1080 cells (**a**–**d**) as showed CLSM using FITC as the fluorescence probe. (**a**) PTX-NPs (**b**) PLNs (**c**) Targeted kNGR-PLN-NPs in HT-1080 (**d**) kNGR-PLNs in the presence of excess kNGR in HT-1080 cells. Microscopic images were taken as 20X magnification.

Because PTX inhibits cell division during the G2/M phase, the G2/M phase rise and G2/M phase seizure suggest cell division inhibition and cell growth restraint [37]. Flow cytometry was used to investigate the impact of several PTX-based PLNs on the cell cycle in HT-1080 cells. As shown in Figure 6, treatment of HT-1080 cells with PTX formulations for 36 h induced a G2/M phase arrest of the cell cycle significantly. The percentage of cells with the G2/M phase was increased from 9.92 ± 0.78 (for the control group) to 20.17 ± 1.23 (for PLNs-kNGR). In the sub-G0/G1 phase, the DNA content in PTX-NPs, PLNs, and PLNs-kNGR is observed at 23.54%, 28.16%, and 34.54%, respectively, at 36 h after treatment with various PTX-based N.P.s, respectively (Figure 6A,B).

### 3.7. Cellular Uptake of N.P.s and Competition Assay

The qualitative cellular uptake of different FITC-loaded N.P.s in HT-1080 cells was further detected using fluorescent microscopy, as shown in Figure 6C. The various cells treated with another category of PLNs displayed fluorescence analogous to the type of formulation. The results show that PLNs-kNGR exhibited higher fluorescence intensity due to kNGR functionalization particles, considerably aiding the N.P.s uptake by HT-1080 cells (Figure 6C(c,d)). The presence of free kNGR peptide competitively inhibits the uptake of kNGR-modified PLNs to a level lower than the nonspecific cellular uptake of unconjugated PLNs (Figure 6e,f), which further confirmed that the uptake of PLNs-kNGR was specifically done by the conjugation of peptide and CD13 proteins over-expressed on HT-1080 cells.

### 3.8. In-Vivo Antitumor Activity

**Figure 7:**
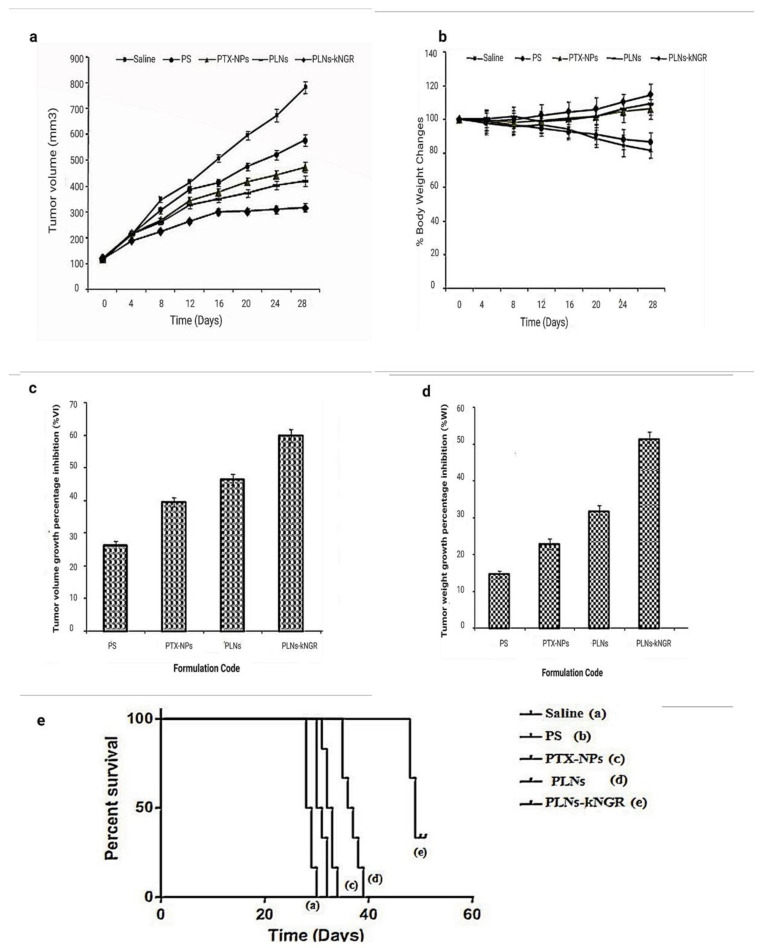
In-Vivo antitumor efficacy of the different PTX formulations in HT-1080 cell-induced tumor in Balb/c mice by changes of (**a**) tumor volumes (**b**) % body weight changes of Balb/c mice by bearing HT-1080 tumor cells (**c**) the effect of free PTX and PTX-loaded formulations on volume growth percentage inhibition (% VI) of different treatments (**d**) The effect of various formulations on tumor weight growth percentage inhibition (%WI) of different treatments on growth of established HT-1080 tumor-induced Balb c/mice at the end of therapy (**e**) Kaplan–Meier survival curves of HT-1080 cell-bearing mice treated with different PTX formulations.

In-vivo antitumor activity of PTX-based formulations was evaluated in Balb/c mice bearing HT-1080 tumor cells. As seen in Figure 7a, all treatment groups slowed tumor development compared to the control group; nevertheless, tumor sizes varied significantly across groups. For example, the tumor volume of PTX-NPs, PLNs, and PLNs-kNGR was 1.22-fold, 1.38-fold, and 1.83-fold smaller than the P.S.-treated group (Figure 7a).

The body changes were used as one of the markers for safety. As shown in Figure 7b, the bodyweight of mice treated with various PTX-based N.P.s increased. On the other hand, the P.S.-treated group showed a serious decrease in body weight (13.4%) due to its lethal side effects [38].

The tumor volume of the PTX-treated groups was significantly lower than the saline group after 28 days, and they followed the sequence: PLNs-kNGRs < PLNs < PTX-NPs < P.S. < saline. The tumor volume growth percentage inhibition (%VI) of PLNs-kNGR, PLNs, PTX-NPs, and P.S. were calculated to be 59.7%, 46.4%, 39.5%, and 26.2% (Figure 7c), while tumor weight growth percentage inhibition (% W.I.) treated with PLNs-kNGR was 2.25-fold higher than those of PTX-NPs (Figure 7d). The antitumor efficacy of the ligand-anchored formulation was superior to that of other nanoparticulate systems or free PTX injection in the mice model.

Kaplan–Meier survival curves’ survival experiment assay was performed on tumor-induced Balb/c mice (Figure 7e). The median survival time of mice treated with kNGR ligand-targeted PLNs-kNGR (49 days) was significantly extended than with physiological saline (28 days, *p* < 0.001), free drug solution (P.S.) (32 days, *p* < 0.001), and PTX-NPs (33 days, *p* < 0.001) and PEGylated PLNs (37 days, *p* < 0.001) through log-rank analysis, respectively (Table 3).

### 3.9. Biodistribution Studies and In-Vivo Toxicological Parameters

The curves showing blood clearance for PTX-loaded N.P.s after i.v administration to Balb/c mice are shown in Figure S7. After 48 h, the % recovered dose in plasma for PTX-based nanoparticulate formulations was 0.54 ± 0.03 and 1.56 ± 0.17 for PLNs and PLNs-kNGR, respectively. No PTX was identified in the case of PTX-NPs after 24 h.

Figure S8 shows the biodistribution characteristics of the different formulations in organs up to 48 h after injection. The accumulation of the PTX-NPs, PLNs, and PLNs-kNGR within the tumors was 4.5, 21.4, and 23.9 times higher than that of the P.S.-treated group at 24 h post-injection, respectively (Figure S8a) (Detailed information found in supplementary file). Free PTX showed a noticeable reduction in blood cell count due to direct exposure to blood cells for the designated period. The hematological toxicity of several nanoparticulate formulations (such as PTX-NPs, PLNs, and PLNs-kNGR-NPs) was reduced (Table S1). The serum urea and creatinine level elevation were much less in animals administered with PLNs-kNGR-NPs formulation. The PLNs-kNGR-NPs (SGOT 12.8 ± 2.9 IU/L; SGPT 14.8 ± 2.6 IU/L; ALP 71.4 ± 6.4 IU/L concentration) revealed an insignificant change in the activity of enzymes compared with control animals (Table S2).

## 4. Discussions

**Figure 8:**
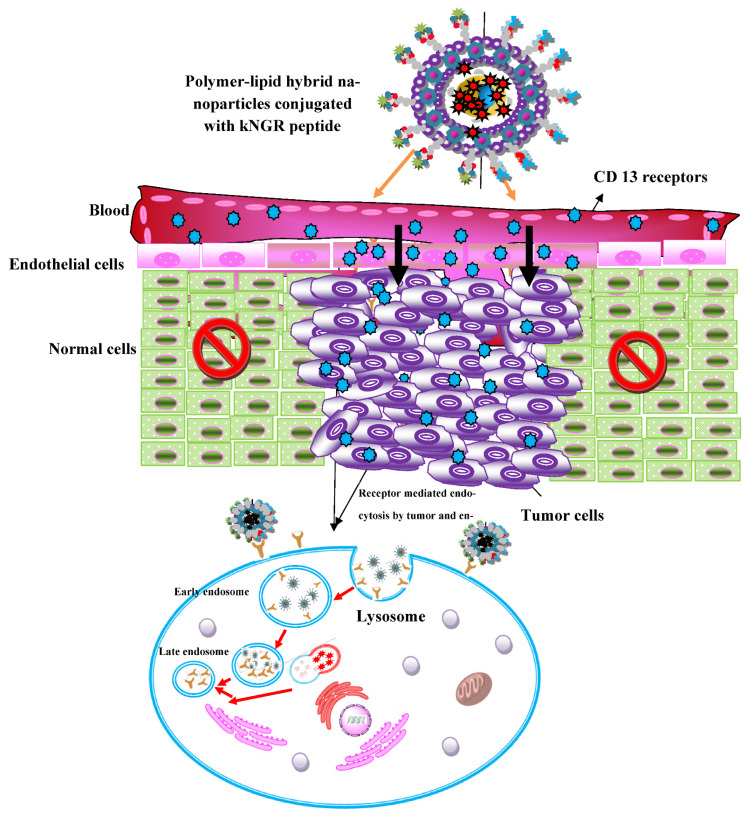
Schematic diagram showing the ligand-conjugated NPs for dual-targeted delivery of solid tumor cells and tumor endothelial cells.

The progression of solid tumors has critically depended on getting adequate blood supply by newly generated blood vessels. However, the proliferation of the endothelial cells contributed to tumor angiogenesis. A previous study reported that among various receptors, CD13 receptors are overexpressed on tumor angiogenic blood endothelial cells, as well as in tumor cells, and play an important role in tumor angiogenesis, invasion, and metastasis. It has been demonstrated that NGR peptides could bind to the CD13 receptors at their presentation site. The kNGR is a novel peptide made up of 5 amino acids (KNGRG) and presents higher tumor-targeting capability. Here, a novel dual-targeting polymer–lipid hybrid nanoparticle was developed by conjugating with kNGR peptide, which was specifically bound to CD13 receptors and delivered the drug at the targeted site [39]. The ex-vivo and in-vitro findings reveal that PLNs-kNGR enhanced cellular uptake in HT-1080 cells and HUVEC cells and their higher accumulation in HT-1080 cell-bearing tumor-induced mice in-vivo. Nanoparticles conjugated with kNGR peptide resulted in a larger particle size than unconjugated nanoparticles, due to the outer orientation of the kNGR peptidic moiety. In addition, peptidic moiety resulted in the slightly more negative zeta potential of ligand-conjugated PLNs than unconjugated PLNs. The PEG group facilitates the presentation of the carboxylic acid on the N.P.s’ surface (Figure 8) [40].

The in-vitro release profile supported the initial fast release of surface-associated PTX. In contrast, the sustained and controlled release depended on drug diffusion from within the inner core of hydrophobic PLGA. The lipid layer on the outer region of PLNs acts as a boundary marker. It constrains water molecules away from the core, preventing hydrolysis and erosion, ultimately slowing down drug release. The drug release results suggest that PTX molecules remain encapsulated during circulation, causing lower systemic toxicity. Furthermore, drug release was increased in acidic tumor environments and intracellular organelles, owing to pH sensitivity [41].

The cytotoxicity of the free drug and PLNs-NPs was assessed using HT-1080 cells and HUVEC cells [42,43]. The results indicate that the targeted system presented maximum cytotoxicity effect, rather than HUVEC cells, due to the higher density of CD13 receptors in HT-1080 cells [44]. The population-doubling rate of HUVEC cells is 30 h (longer cell cycle), while HT-1080 doubles in 18 hrs. I and thus PTX-PLNs are more sensitive to HT-1080 cells. The apoptosis study strongly demonstrated that among the various PTX-based N.P.s, the PLNs-kNGR is the most efficacious formulation due to higher apoptosis in tumor cells [45]. The MTT and cell apoptosis assay confirmed that HT-1080 tumor cells were more sensitive to PTX-based formulations [46].

The cell cycle analysis strongly demonstrated that among the various PTX-based NPs, the PLNs-kNGR is the most efficacious formulation that induced cell growth arrest. The data reveal that the enhanced cytotoxicity observed for targeted PLNs results from an enhanced intracellular PTX concentration due to their receptor-mediated endocytosis and confirmed their superior antitumor activity. Hence, the findings demonstrate that kNGR peptide-mediated endocytosis of PTX-loaded PLNs play a pivotal role in PTX-induced apoptosis and cell arrest in the G2/M phase [47]. Thus, the results show that cell cycle arrest was formulation-dependent. Furthermore, the cellular uptake of PLNs in HT-1080 cells was enhanced by conjugating kNGR peptidic moiety. The results also state that the internalization of PLNs-kNGR was ligand-dependent; in addition, pre-conditioning of cells with kNGR peptides results in reduced uptake, as CD13 receptors were competitively binding with kNGR peptide. To verify the dual-targeting effects of PLNs-kNGR in-vivo, HT-1080 cell-induced tumor-bearing Balb/c mice were used. The PLNs show a greater tumor growth inhibitory effect than free PTX. Enhanced accumulation through receptor-mediated uptake in tumor cells may be linked to higher anticancer efficacy of the targeted PLNs [48].

Furthermore, the results indicate that the targeting ligand kNGR could markedly improve the antitumor effect of the PLNs-NPs. Compared to other treated groups, the %VI and % W.I. was considerably greater after 28 days of experimental treatment due to the superior antitumor efficacy of ligand-anchored formulations. Furthermore, compared to other nanoparticulate systems, the PLNs-kNGR-treated group had considerably longer animal survival times because of the enhanced concentration of PTX in the tumor tissue via the EPR effect and ligand–receptor interaction [49].

The plasma concentration profile of PLNs and PLNs-kNGR indicated greater in-vivo stability of the formulations, as the formulation has a longer systemic circulation time. This suggests that nanoparticulate systems had slowed the drug release almost in a sustained manner compared to other formulations [50]. The tissue biodistribution studies show that the different extent in tumor PTX disposition was formulation design-dependent. The targeted kNGR peptide-anchored PLNs presented maximum drug accumulation in tumor tissue due to ligand–receptor interaction. Previous studies supported that combination of passively targeted systems and actively targeted systems may alter the biodistribution pattern of the encapsulated drug remarkably with enhanced antitumor efficacy.

The overall study findings reveal that kNGR peptide-mediated lipid–polymer-based nanoparticles play an important role for targeting the tumor cells, as well as tumor endothelial cells. Literature cited that CD13 receptors are overexpressed by both tumor cells and tumor endothelial cells. Our ex-vivo studies also support that designed kNGR-conjugated nanoparticles specifically bound to CD13 receptors and delivered the drug at the targeted site and enhanced the therapeutic efficacy.

## 5. Conclusions

The present study proposed and synthesized successfully engineered dual-targeting PLNs-kNGR to augment the intracellular delivery of hydrophobic drugs, i.e., PTX. It was concluded that kNGR-functionalized PLNs facilitated the intracellular delivery retention of loaded PTX in HT-1080 cells and HUVEC cells that over-expressed CD13 receptors and enhanced cytotoxicity and G2/M phase arrest well. Furthermore, the in-vivo study further exhibited a higher survival period for tumor-induced mice in the case of PLNs-kNGR. As a result, the current formulation of polymer–lipid hybrid N.P.s functionalized with kNGR has a strong potential to be a more effective dual-targeting drug carrier(s) for treating solid tumors.

## Figures and Tables

**Table 1:** Physicochemical characteristics of various PTX-loaded NPs.

Formulation Code	Size (nm)	PI (Polydispersity Index)	Zeta Potential (mV)	%EE (Entrapment Efficiency)	CE% (Conjugation Efficiency)	Surface Density (P)	(Average Distancein nm) D
PTX-NPs (Polymer-based nanoparticles)	163.5 ± 5.52	0.128 ± 0.011	−22.4 ± 1.8	72.24 ± 4.43	---	---	---
PLNs (Polymer–lipid hybrid nanoprtaicles without ligand)	178.8 ± 8.41	0.126 ± 0.012	−26.6 ± 1.9	78.88 ± 5.38	---	---	---
PLNs-kNGR (Ligand-conjugated Polymer–lipid hybrid nanoprtaicles)	205.1 ± 9.1	0.117 ± 0.011	−31.3 ± 2.3	82.21 ± 3.75	34.7	198 ± 6.8	16 + 1.4

**Table 2:** IC_50_ value of various PTX-loaded formulations on HUVEC and HT-1080 cell lines following 24, 48 and 72 h treatment, respectively.

Time (h)	HUVEC Cell Line, IC_50_ μg/mL)				HT-1080 Cell Line, IC_50_ (μg/mL)			
	PS	PTX-NPs	PLNs	PLNs-kNGR	PS	PTX-NPs	PLNs	PLNs-kNGR
24	22.5	23.5	18.8	9.6	18.5	19.8	12.9	7.6
48	19.3	13.8	8.9	3.3	17.2	9.0	5.6	2.4
72	12.1	7.2	2.72	0.98	9.25	4.8	2.2	0.85

**Table 3:** The effect of free PTX drug and PTX-loaded PLNs on tumor volume growth inhibition (%VI), tumor weight growth percentage inhibition (%WI), and survival time of different treatments on growth against HT-1080 tumors in Balb c/mice at the end of therapy. ns = not significant, ** *p* < 0.01, *** *p* < 0.001.

Formulation Code	Dose (mg/kg)	%VI	%WI	Median Survival Time (days)	Mean Survival Time (days)	Standard Error	95% Confidence in Interval	Increase in Survival Time (% IST)	Log Rank Test			
									Saline	PS	PTX-NPs	PLNs
Saline	---	---	---	28	28.3	0.516	27.791–28.875	---	---	---	---	---
PS	10	26.2 ± 1.2	14.6 ± 1.0	32	31.7	0.422	30.583–32.751	14.3	** *p* < 0.01	---	---	---
PTX-NPs	10	39.5 ± 1.5	22.8 ± 1.4	33	32.7	0.422	31.583–33.751	17.9	*** *p* < 0.001	ns (*p* >0.05)	---	---
PLNs	10	46.4 ± 1.7	31.6 ± 1.7	37	36.7	0.76	34.712–38.621	32.1	*** *p* < 0.001	*** *p* < 0.001	*** *p* < 0.001	---
PLNs-kNGR	10	59.7 ± 2.1	51.3 ± 1.9	49	49.2	0.477	47.940–50.394	75	*** *p* < 0.001	*** *p* < 0.001	*** *p* < 0.001	*** *p* < 0.001

## Data Availability

The data presented in this study are available on request from the corresponding author.

## Supplementary Materials

The following supporting information can be downloaded at: https://www.mdpi.com/article/10.3390/pharmaceutics14071401/s1.

## Abbreviations

kNGR (Asn-Gly-Arg): NGR based peptide ligand, DSPE-PEG: 1, 2-Distearoyl-sn-glycero-3-phosphoethanolamine-Poly(ethylene glycol), PTX: Paclitaxel, PLNs-kNGR-NPs: PLGA–lecithin–PEG core-shell nanoparticles, PLNs: polymer lipid hybrid nanoparticles, PLGA: Poly(lactic-co-glycolic acid), DOX: Doxorubicin, HUVEC: Human endothelial cell line, HT-1080: Human fibrosarcoma cell line, FITC: Fluorescein isothiocyanate, PI: Propidium iodide, MTT: 3-(4,5-dimethyl thiazol-2-yl)-2,5-diphenyltetrazolium bromide, NHS: N-Hydroxysuccinimide, DCC: N, N′-Dicyclohexylcarbodiimide, DMSO: Dimethyl sulfoxide, PCS: Photon correlation spectroscopy, TPVG: Trypsin Phosphate Versene Glucose, % EE: Percentage entrapment efficiency, FBS: Fetal bovine serum, BSA: Bovine Serum Albumin, PS: Paclitaxel based drug solution, % VI: Percentage volume growth percentage inhibition, %WI: Percentage tumor weight growth percentage inhibition etc.
