# Supplementary material for: A kNGR Peptide-Tethered Lipid–Polymer Hybrid Nanocarrier-Based Synergistic Approach for Effective Tumor Therapy: Development, Characterization, Ex-Vivo, and In-Vivo Assessment
Source: Pharmaceutics. 2022 Jul 3;14(7):1401. doi: 10.3390/pharmaceutics14071401 (PMC9320317; doi:10.3390/pharmaceutics14071401)
Supplement: Supplementary file 1 [file pharmaceutics-14-01401-s001.zip › pharmaceutics-1764823-supplementary.pdf]

Supporting information's

kNGR-peptide tethered lipid-polymer hybrid nanocarriers based synergistic approach for effective tumor therapy: Development, Characterization, *ex-vivo* and *In-vivo* assessment

Materials and methods

Materials

Paclitaxel and Poly-lactide/glycolide (PLGA) were gifted by Sun Pharma lab, Surat, India. kNGR peptide was synthesized and purchased from USV Ltd. Mumbai, India. 1,2 distearoyl-sn-glycero – 3 – phosphoethanolamine – N –[carboxy (polyethylene glycol)-200] was procured from Avanti, USA. Fluorescein isothiocyanate (FITC), DNAse-free RNAse, MTT and Propidium iodide were procured from Sigma Aldrich, USA.

Synthesis and characterization of DSPE–PEG–kNGR conjugate

DSPE–PEG–kNGR conjugate was synthesized using kNGR peptide and DSPE-PEG$_{2000}$-COOH with the previously reported method [5]**.** FT-IR was used to characterize the synthesized DSPE–PEG–kNGR.

Preparation and characterization of polymer lipid hybrid NPs (PLNs)

PTX-loaded PLNs were created as a slight variant on a previously published technique. To develop PLGA–lecithin–PEG core–shell PLNs, PLGA, egg lecithin (PC), and DSPE–PEG were combined utilising a modified nanoprecipitation technique and self-assembly. To begin, the organic phase was prepared by dissolving PLGA and PTX in acetonitrile in weighed proportions. At a concentration of 4% ethanol, DSPE-PEG COOH (molar ratio 5:1) and PC (molar ratio 5:1) were dissolved in water (at polymer weight 20 percent). The aqueous phase of unconjugated PLNs is composed of PC, DSPE–PEG2k (22). The warmed aqueous phase (65oC) was gently mixed before to adding the organic phase dropwise (1 ml/min) under vortexing for 3 minutes. Over the course of two hours, the organic solvent was allowed to evaporate while the NPs were regularly stirred. The solution was purified three times using an Amicon

Ultra-4 centrifugal filter with a MWCO of 10 kDa, after which it was resuspended in water. Sterilization of the dispersion was accomplished using a 0.22m filter unit and storage at 4oC [24].

A scanning electron microscope (SEM) was used to examine the PLNs' morphology. Zetasizer nano ZS90 PCS was used to evaluate the average particle size, PI and zeta potential at 25°C. After dissolving a predetermined number of PLNs in 1 ml of acetonitrile and gently shaking for 5 minutes, samples were sonicated to determine the percentage of effective encapsulation (EE). After centrifugation (at 25,000 rpm for 30 minutes on a Hitachi CPMax-100, Japan), an HPLC system was used to determine the concentration of medication in the supernatant. An HPLC system with a Hypersil C18 column (250x4.6mm, 5) and a Waters C18 Nova-Pak guard column (20x3.9mm, 5) was used to find out the concentration of the toxin PTX. Mobile phase was acetonitrile: methanol: ammonium acetate buffer solution (50:20:30 v/v/v) with flow rate 0.8 ml/min and detection wavelength of 227 nm.

Determination of cNGR conjugation efficiency and density on NPs surface

The CBQCA Protein Quantitation Kit was used to determine the kNGR concentrations in this experiment (Molecular Probes). We were able to calculate the average distance (d) and average conjugation efficiency (CE percent) between two neighbouring PEG chains using previously described techniques.

In-vitro drug release

Phthalate buffer solution (PBS, pH 7.4) and phosphate buffer solution were used as release media in the in vitro drug release study, as reported before. A 30 ml release medium was dialyzed against 2 ml of PLNs and dialyzed at 37°C and 100 rpm with slow but continuous magnetic stirring for 30 minutes. The aliquots from the release media were collected and replaced with new media at regular intervals. Samples were obtained in triplicate for each data point and the drug content was tested three times.

Evaluation of protein adsorption to PLNs surface

An earlier approach, somewhat modified, was used to measure protein adsorption on the surface of PLNs. PLNs were cultured for 24 hours at 37°C in either 5 percent FBS or 2 percent bovine serum albumin before being evaluated by PCS with the Zetasizer nano ZS90 [3].

Hemolysis toxicity

In accordance with the previously stated approach, the hemolytic toxicity was carried out. This article included a supplemental file that explained the technique in great detail. HiAnticlot blood collection vials were used to collect a 5 percent hematocrit RBC suspension from human blood. Separately, 0.5 ml of drug solution and 4.5 ml of normal saline were used to incubate RBC suspension with appropriately diluted drug solution and nanoparticulate systems. After centrifuging for 15 minutes at 2500 rpm, the tubes were left to stand for 1 hour with mild intermittent shaking. After centrifugation, normal saline was used to dilute the supernatants. The degree of hemolysis was assessed by measuring the absorbance at 540 nm [7].

Trypan blue exclusion assay and clonogenic assay

The previously reported Trypan blue exclusion test was used. HUVEC and HT-1080 cells were plated in a well with 1ml of DMEM medium and incubated overnight at 37°C, 5% CO2, and 95% air. Samples were taken after 8, 12, 24, and 48 hours of incubation in sterile polypropylene tubes following treatment with different drug-loaded NPs. The cells were then incubated for 5 minutes with 0.4 percent trypan blue in 0.9 percent saline before being counted with a haemocytometer to determine whether or not they were viable [25].

According to prior research, the anticancer activity of nanoparticulate systems was tested. In accordance with previously described procedures, the anticancer activity of nanoparticulate systems was investigated. Samples were incubated with the cell cultures for 48 hours after they had been seeded with 5105 HT-1080 and HUVEC cells. A growth media was used to incubate the cells, which had been treated

with trypsin, in petridishes containing 10,000 cells each. Colonies of viable cells were fixed and stained with 0.5 percent methylene blue solution in ethanol for 10 to 14 days, and the plating efficiency (PE) was measured.

Evaluation of cell apoptosis activity

$2x10^5$ cells per well were seeded into 6-well plates and grown for 24 hours at 37°C with a cover slip. Using the formulations and culture media as a control, cells were cultured for a further 36 hours. After that, samples were fixed in PBS containing 4% paraformaldehyde for 15 minutes before being stained in PBS containing 0.2 g/ml DAPI for 15 minutes before being rinsed twice in ice-cold PBS. Glass slides have cover slips attached to them. The Olympus 170 inverted fluorescence microscope was used to view the slides.

Cytotoxicity assay

Both HT-1080 and HUVEC cells, seeded at a density of 5000 cells per well in a 96-well plate containing DMEM media and cultured for 24 hours, were used in the cytotoxicity experiment. 100 l of new medium was then added and incubated for 24, 48, and 72 hours with different formulations at a concentration of 2.5-30 g/ml. MTT (5 mg/ml) was added to DMEM and the cells were cultured for an additional four hours at 37°C before the media was changed back to MEM. Finally, the MTT solution was withdrawn from the wells and each well received 100 l DMSO to dissolve any precipitated formazan crystals. Before using a microplate reader to measure optical absorbance at 540 nm, the plate was shook vigorously for 15 minutes.

***In-vivo* study**

Biodistribution studies

A number of PTX-loaded formulations were tested on tumour generated Balb/c mice for their biodistribution investigations. Each of the 27 animals in each group was randomly assigned to a different group. Ten

milligrammes of PTX per kilogramme of body weight was administered to each group. The mice were slaughtered after receiving i.v. injections at predetermined intervals (0.5, 1, 2, 4, 6, 8, 12, 24 and 48 hours). Samples of blood and other organs were taken from the retro-orbital plexus at various times in time (kidney, spleen, heart, lung, liver, and tumor). They were then rinsed in normal saline and kept at -20°C until they could be examined using an HPLC system. In order to assess the clearance and biodistribution of formulations, PTX was extracted into acetonitrile and HPLC-analyzed for the presence of the drug in the blood serum and various organs [27].

***In-vivo* toxicological parameters**

Hematological study

The haematological parameters of Balb/c mice were measured. Three mice were placed in each of the three treatment groups. A total of 10 mg/kg of PTX or saline was administered intravenously to each group once every two days throughout the course of a week. RBC, WBC, platelets, and haemoglobin (Hb) concentrations were measured in a pathology lab 24 hours after the last delivery of the drug in all groups [30].

Evaluation of nephrotoxic and hepatotoxic effect

Three Balb/c mice were placed in each of the different treatment groups. Each group got an intravenous injection of 10 mg/kg body weight nanoparticulate systems. Blood samples were taken from the eye's retro-orbital plexus 72 hours after various formulations were administered. Serum levels of urea, creatinine, SGOT, AST and SGPT were measured after the samples were centrifuged at 3500 rpm for 20 minutes [30].

Results and discussion

Synthesis and characterization of DSPE–PEG–kNGR conjugate

The kNGR peptide was conjugated to the carboxyl group of DSPE-PEG-COOH by NHS and DCC approach to enhance specificity and targeting effectiveness against overexpressed CD13 receptor on HT-1080 and HUVEC cells. The in-depth investigation found in the supplemental file (Figure 1).

When the product was examined using FT-IR, significant peaks for DSPE–PEG–kNGR were observed at 1745 cm1 (ester conjugated C=O), 2851 and 2929 cm1 (aromatic C=C bending and stretching), 807.5 cm1 (aromatic C-H bending), 3327 cm1 (N-H stretch of primary amino and amide), and 1418 cm1 (C-N stretching) (Figure suppl.1).

Preparation and characterization of polymer lipid hybrid NPs (PLNs)

Unconjugated and kNGR-anchored PLNs may be prepared using the modified nano-precipitation approach. Nanoprecipitation was used to create the self-assembled PLNs dispersion with a hydrophobic PLGA core, a hydrophilic polymer (DSPE–PEG) shell, and a lecithin monolayer at the interface of the PLGA core and PEG shell (Figure 2A).

Assembled on the hydrophobic PLGA core, PC and DSPE–PEG2000 phospholipids (DSPE–PEG2000) were found to form the aqueous phase. Solubility, integrity, and diffusion barrier are all provided for encapsulated pharmaceuticals with the use of this formulation. A monolayer of lipids is dispersed around the PLGA core to enhance thermal and mechanical energy. RES absorption and aggregation are inhibited by the hydrophilic PEG on the PLNs, which serves as a linker for surface conjugation.

SEM images of PLNs-kNGR showed a smooth, spherical morphology with nanometer-sized dimensions (Figure 2B). Table 1 summarises the NPs' physicochemical properties. Comparing ligand-conjugated PLNs to unconjugated PLNs with smaller sizes and lower EE percentages, the mean diameter of ligand-conjugated PLNs was larger at 205 nm. As expected, ligand-conjugated PLNs had an increased zeta potential due to the nanoparticles' negative charges being dispersed more evenly over their surface area.

On the NPs surface, the surface density was 198 and the kNGR peptide conjugation efficiency was 34.7 percent. For the kNGR peptide moiety-based nanoparticles, the average distance between two nearby PEG chains was 16 nm (shown in table 1). To better bind CD13 receptors, this property, which is attributable to the surface-attached multivalent array of the kNGR-peptidic ligand, would be beneficial.

In-vitro release study

Drug-loaded PLNs must have a regulated and prolonged drug release. Due to hypoxia and acidic intracellular organelles, tumour tissue has a lower pH than normal tissue, which has previously been described. Consequently, an in-vitro drug release research was conducted at 37°C in a simulated physiological state (pH 7.4) and in an acidic environment (pH 5.0). In accordance with figure suppl.2, the PLNs and PLNs-kNGR showed an early burst release followed by a persistent release of drugs from NPs at pH 7.4 and pH 5.0, respectively. At pH 7.4 (figure suppl. 2a), 27.7 percent of PTX from kNGR-oriented PLNs was released, whereas 46.5 percent of the drug was released from PLNs-kNGR at pH 5.0 in 24 hours (figure suppl.2 b). Within 15 days, 75.6% of the medication was released in PBS pH 7.4 and 90.5% was released in pH 5.0 using target-oriented PLNs-based nano-particulate systems in the next 9 days.

Plasma protein adsorption

The phagocytosis absorption was made easier by the plasma protein adsorption on the particle surface. Both PLNs were incubated in the presence of 5 percent FBS, followed by 2 percent BSA, for a total of 10 minutes. Practically all studied PLNs remained nearly unaltered in their average diameter prior to FBS incubation (figure suppl.3 a,b). After 24 hours of incubation with 5 percent FBS, the particle size of PLNs and PLNs-kNGR remained essentially unaltered. After 24 hours of incubation with 5% FBS in the presence of PTX-NPs (simple PLGA-NPs), the NPs were bigger and had a wider PI. Co-incubation of NPs also had similar effects (figure. suppl.3 c, d). Due to the steric hindrance of the crown polymer (PEG), the adsorption of plasma protein is not permitted, and therefore aggregation may be avoided.

Hemolytic toxicity

Maximum drug concentration of the free drug solution resulted in 38.4 percent hemolysis. At greater drug concentrations, the drug-loaded plain PLGA-NPs (PTX-NPs) hemolyzed 28.2%. The hemolytic toxicity of PLNs-NPs, on the other hand, was lower than that of simple NPs. At all doses, ligand-conjugated NPs, on the other hand (PLNs-kNGR), greatly reduced hemolysis (figure suppl.4). Research shows that kNGR-peptide conjugation on the NPs surface greatly decreased RBC hemolysis by reducing the contact between RBCs. The inclusion of a stealth PEG layer and ligand moiety on the nanoparticulate surface may account for the decreased hemolytic impact seen with unconjugated systems and target-oriented NPs.

Influence of drug loaded NPs formulations on cell membrane integrity

Figure suppl.5 shows the improved membrane integrity of HT-1080 and HUVEC cells after treatment with PTX-based formulations throughout a range of time intervals. PS did not substantially alter the normalised membrane integrity even after 48 hours of exposure, according to all of the data. Only after extended incubation with both cell lines of PLNs-kNGR-NPs was the restored membrane integrity more clearly visible. PLNs-kNGR-NPs were the most toxic and had the greatest impact on membrane integrity degradation of all the PTX formulations tested.

Anticancer activity of various formulations determined by clonogenic assay

The clonogenic test is used to examine the long-term growth suppressive effects of different PTX-based formulations on both cell lines, as shown in figure suppl.6. At concentrations of 2.5 g/ml, PLNs-kNGR based therapies displayed considerably increased toxicity and cell death in HT-1080 cells. It was shown that in the case of HUVEC cells, the PE was 1.54 and 1.34 times lower for PLNs-kNGR based therapy than for the other two treatments. Targeted NPs were more hazardous to both kinds of cells, according to all the evidence.

Evaluation of cell apoptosis activity

The apoptotic morphology of HT-1080 and HUVEC cells treated with various formulations was investigated to see whether PTX-loaded NPs might induce apoptosis. DAPI-stained pictures of HT-1080 and HUVEC cells are shown in figure 3 A and B, respectively. A substantial proportion of cells showed apoptotic characteristics, such as nuclear shrinkage, chromatin condensation, or fragmentation, when co-incubated with PTX-based nanosystems for 36 hours. However, following DAPI labelling, the nuclei of untreated HT-1080 and HUVEC cells show uniform fluorescence with no signs of segmentation or fragmentation. For both kinds of cells, the proportion of apoptotic cells was much greater when PLNs were coupled with cyclic NGR peptides (Figure 3 C).

Cytotoxicity assay

HUVEC (Figure 4 a, b, and c) and HT-1080 (Figure 4 d, e, and f) cells were incubated for 24, 48, and 72 hours to determine the in vitro cytotoxicity of several PTX formulations, including PS, PTX-NPs, PLNs, and PLNs-kNGR. Proliferation of both cell lines was strongly inhibited by all PTX-containing formulations tested. At the same dosage from 2.5-30 g/ml all formulations demonstrated dose-dependent, time-dependent and formulation dependant cytotoxicity against PTX-infected cells Proliferation inhibition of HUVEC and HT-1080 cells was significantly reduced at varied concentrations of PLNs-kNGR. These formulations were shown to be most effective against cellular proliferation when tested using IC50 values obtained 48 and 72 hours later. PLNs-kNGR>PLNs>PTX-NPs> It was discovered that PLNs-kNGR treatment reduced the IC50 value of HUVEC and HT-1080 cells by 1.96 and 1.7%, respectively, after 24 hours, in comparison to that of PLNs (Table 2). PS was shown to be less effective than PTX-NPs in terms of cell viability at 24 hrs.

Biodistribution studies

After being administered intravenously to Balb/c mice, the PTX-loaded NPs' blood clearance curves are shown in Figure suppl.7. There was an initial increase in blood concentrations of the medication, but the drug was swiftly eliminated from the circulation 12 hours after administration of the PTX-loaded NPs. The circulatory clearance of the nanoparticulate system, on the other hand, was significantly delayed. PLNs and PLNs-kNGR nanoparticle formulations had a recovery rate of 0.540.03 and 1.560.17, respectively, after 48 hours, whereas PTX-NPs had a recovery rate of 0% after 24 hours.

After 48 hours, the biodistribution of each formulation is shown on the Figure suppl. 8 graph. PTX concentrations in the tumours of mice given varied formulations were as follows: In this order, PTX-NPs> PTX-NPs>PS. At 24 hours after injection, the PTX-NPs, PLNs, and PLNs-kNGR groups had 4.5, 21.4, and 23.9 times more PTX-NPs and PLNs in their tumours than the PS-treated group (Figure suppl.8 a). All of the nanoparticulate systems accumulated in the liver, which was its primary organ. It was shown that PTX-NPs (from 35.9 percent of injected dosage to 26.4 percent at 48 hr) and PS (from 39.9 percent of injected dose at 30 minutes to 28.2 percent at 48 hours) accumulated simultaneously in the liver. PLNs and PLNs-kNGR-NPs, on the other hand, demonstrated 32.5 percent, 28.9 percent, and 9.3 percent, 10.4 percent, respectively, drug recovery in the first 30 minutes and 48 hours following intravenous injection, respectively (Figure suppl.8 b). After four hours, medication concentrations in the spleen and lungs of mice in the PS and other nanoparticulate systems began to decline (Figure suppl.8 c and Figure suppl.8 d). It was shown that kNGR-decorated PEGylated PLNs-NPs accumulated more in the spleen than unconjugated PEGylated PLNs-NPs, however. Liposomes modified with kNGR were shown to concentrate in the spleen following intravenous injection in a prior investigation. The PTX content in the heart and kidneys of the PS-treated group was greater than in the control group, which may have shed light on the negative effects of the drug (Figure suppl.8 e and Figure suppl.8 f). For all nanoparticulate systems, PTX levels in the heart and kidney were lower, which was predicted to lessen adverse effects.

In-vivo Hematological study

Researchers used haematological testing on blood components to look for harmful effects of the manufactured formulas. WBC count, RBC count, Hb content, and platelet count were reduced following administration of free PTX drug solution for a set amount of time because of the direct exposure of free medication to blood cells (Table suppl.1). Hematological toxicity reduced when the same quantity of medicine was encapsulated in different nanoparticle formulations (such as the PTX-NPs, PLNs, and PLNs-kNGR-NPs).

Evaluation of nephrotoxic and hepatotoxic effect

Renal function is assessed by measuring serum urea/creatinine levels and liver function is assessed by measuring the SGOT/SGPT/ALP levels. Administration of free medication solution dramatically raised serum urea and creatinine levels (Table suppl.2). Despite this, mice given the PLNs-kNGR-NPs formulation had significantly lower urea and creatinine levels in their blood.

Hepatotoxicity was assessed in vivo by measuring SGOT, SGPT, and ALP concentrations in response to anticancer medication administration in free or entrapped form. An increase in the concentrations of SGOT, SGPT, and ALP in the blood of animals exposed to free PTX resulted in an increase in their toxicity. PTX-NPs, on the other hand, had a smaller effect on SGOT (16.63.1IU/L), SGPT (22.64.7 IU/L), and ALP (80.67.4 IU/L) activity. In comparison to PTX-NPs, the activity of all biochemical measures was lower in PLNs-NPs than in PTX. There was only a little difference in enzyme activity between PLNs-kNGR-NPs and control animals in terms of SGOT (SGOT 12.82.9 IU/L; ALP 71.46.4 IU/L) and SGPT (SGPT 14.82.6 IU/L) (Table suppl.2).

Conclusion

The present study proposed and synthesized successfully engineered dual-targeting PLNs-kNGR-NPs that is anchored with kNGR peptide to augment the intracellular delivery of hydrophobic drug. For solid

tumour therapy, this new lipid polymer hybrid nanoparticle formulation adorned with targeting ligand shows great potential as a more effective opportunistic dual drug carrier.

Table S1: Involvement of several PTX-based nanoparticulate formulations in the measurement of various haematological parameters

Formulations code	WBC (×10³ µl)	RBC (×10⁶ µl)	Platelet count (×10³ µl)	Hb (g/dl)
**Saline**	7.2±0.78	9.8±0.67	1067±128	12.8±1.3
**PS**	5.3±0.48	7.4±0.73	780±120	7.3±0.94
**PTX-NPs**	5.9±0.56	7.7±0.65	862±124	8.5±1.1
**PLNs**	6.4±0.72	8.6±0.92	948±127	9.6±1.5
**PLNs-kNGR**	7.4±0.85	9.3±0.87	1021±117	13.1±1.3

Table S2: Mice treated with free PTX solution and PTX-loaded formulations had varying effects on kidney and liver function measures

Formulations code	Urea (mg/dl)	Creatinine (mg/dl)	SGOT (units/l)	SGPT (units/l)	ALP (units/l)
**Saline**	32.8±2.3	1.17±0.05	12.8±2.1	15.2±3.4	72.6±6.6
**PS**	57.8±5.5	1.74±0.07	23.3±4.1	27.2±4.9	87.3±7.8
**PTX-NPs**	51.1±5.2	1.65±0.07	16.6±3.1	22.6±4.7	80.6±7.4
**PLNs**	45.5±4.4	1.23±0.06	15.1±3.1	19.5±3.6	77.4±6.5
**PLNs-kNGR**	33.4±2.7	1.16±0.04	12.8±2.9	14.8±2.6	71.4±6.4

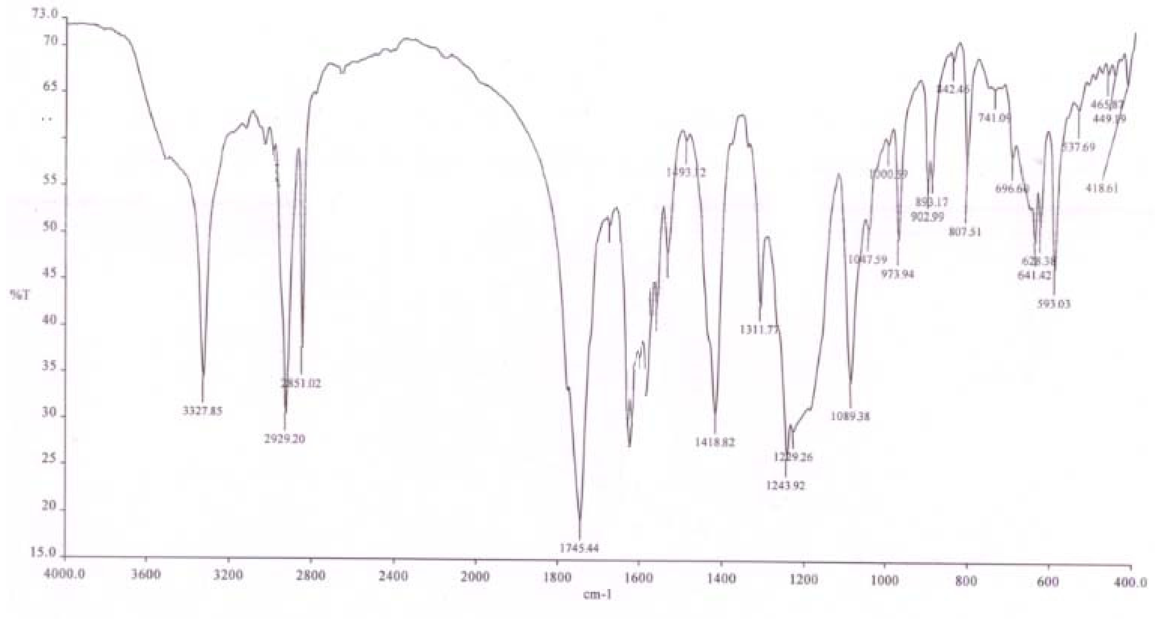

Figure S1: FT-IR spectrum of DSPE-PEG-kNGR conjugation

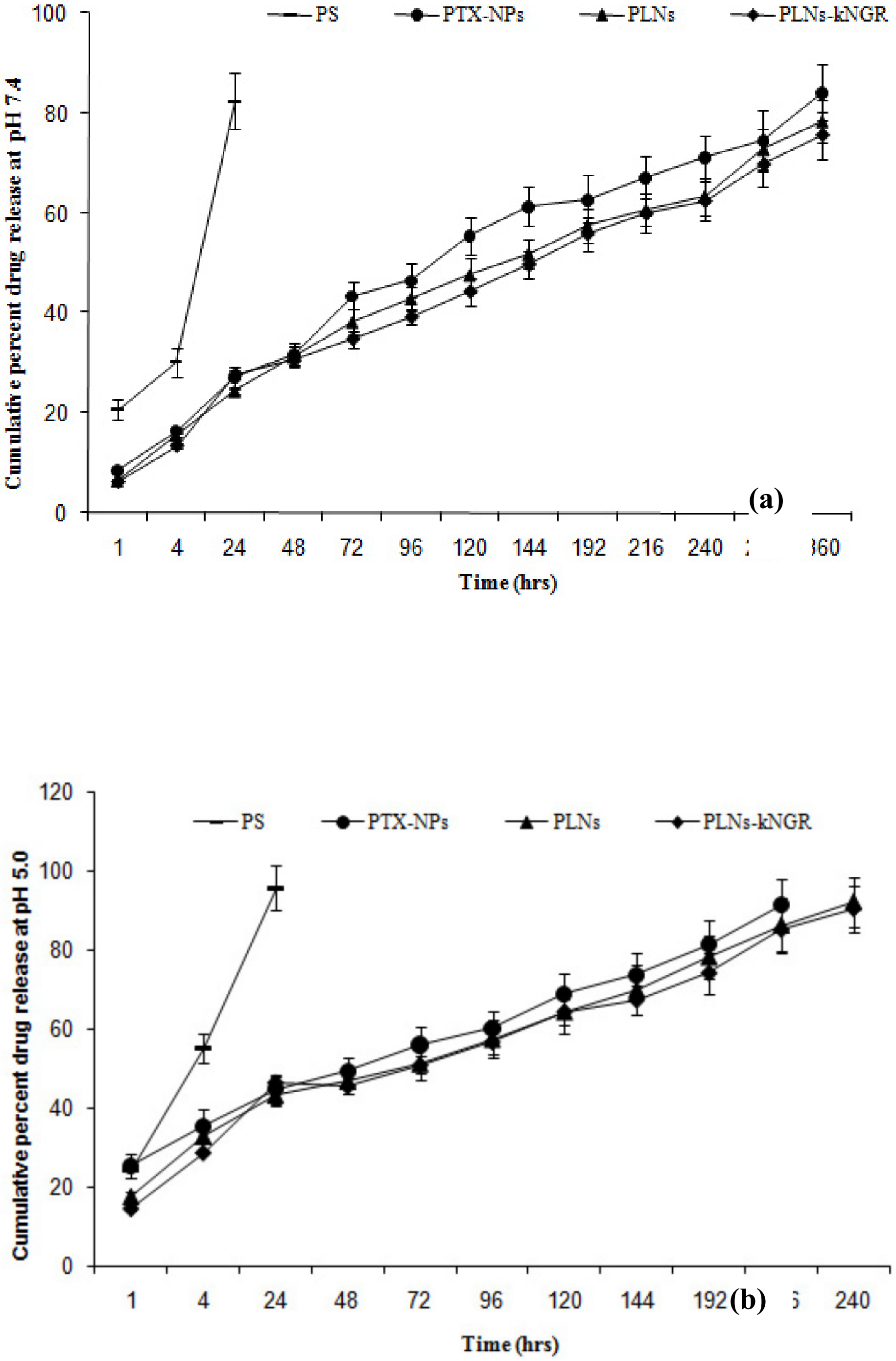

Figure S2: PTX drug release profiles from NPs in vitro at 37±1°C in PBS solution at (a) pH 7.4 (b) phthalate buffer at pH 5.0. Data presented as mean ± SD (n = 3).

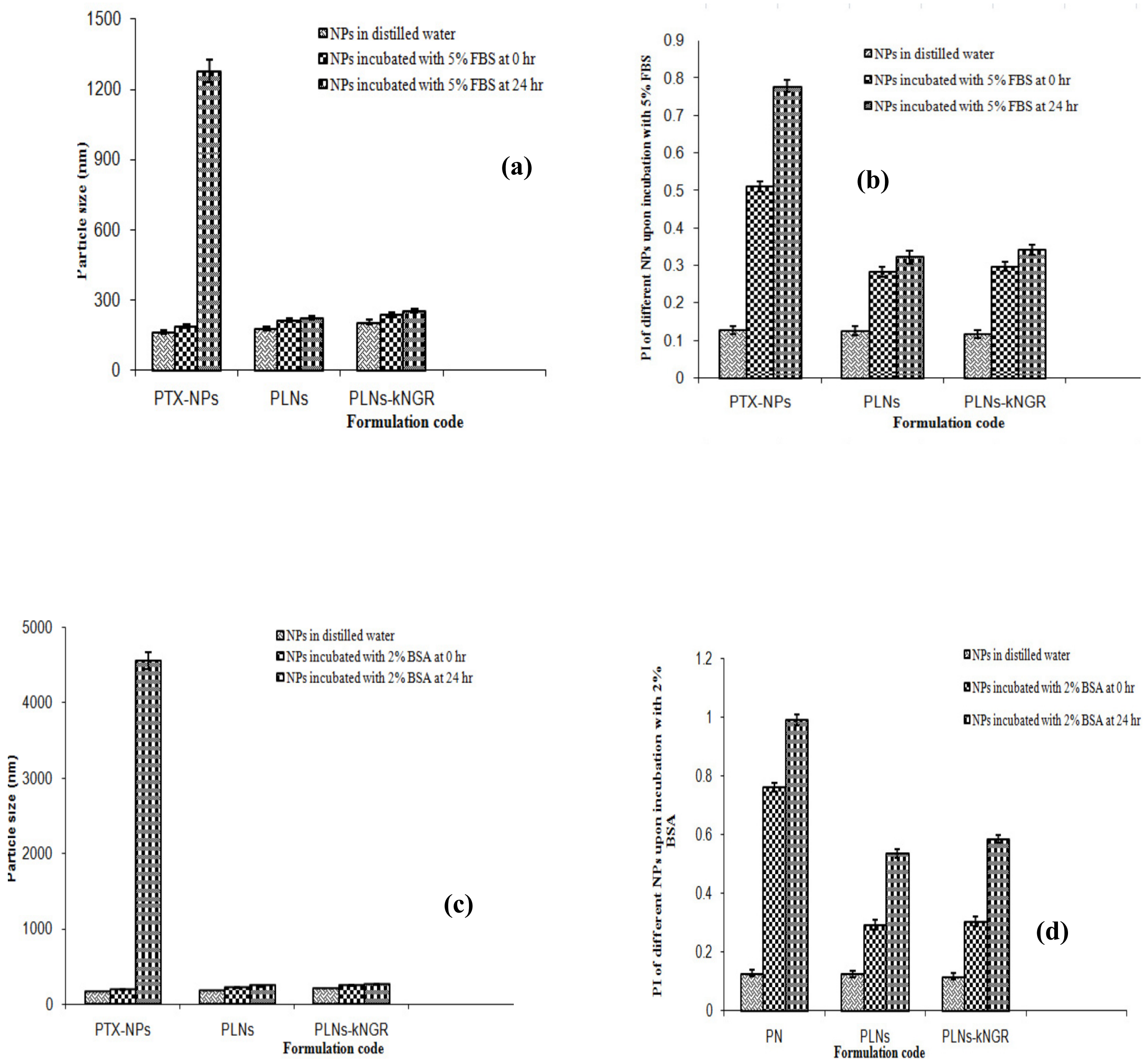

Figure S3: Plasma adsorption studies of different PTX loaded NPs upon incubation at 37◦C for 24 hr with 5% FBS (a) particle size (b) PI; and in 2% BSA (c) particle size (d) PI

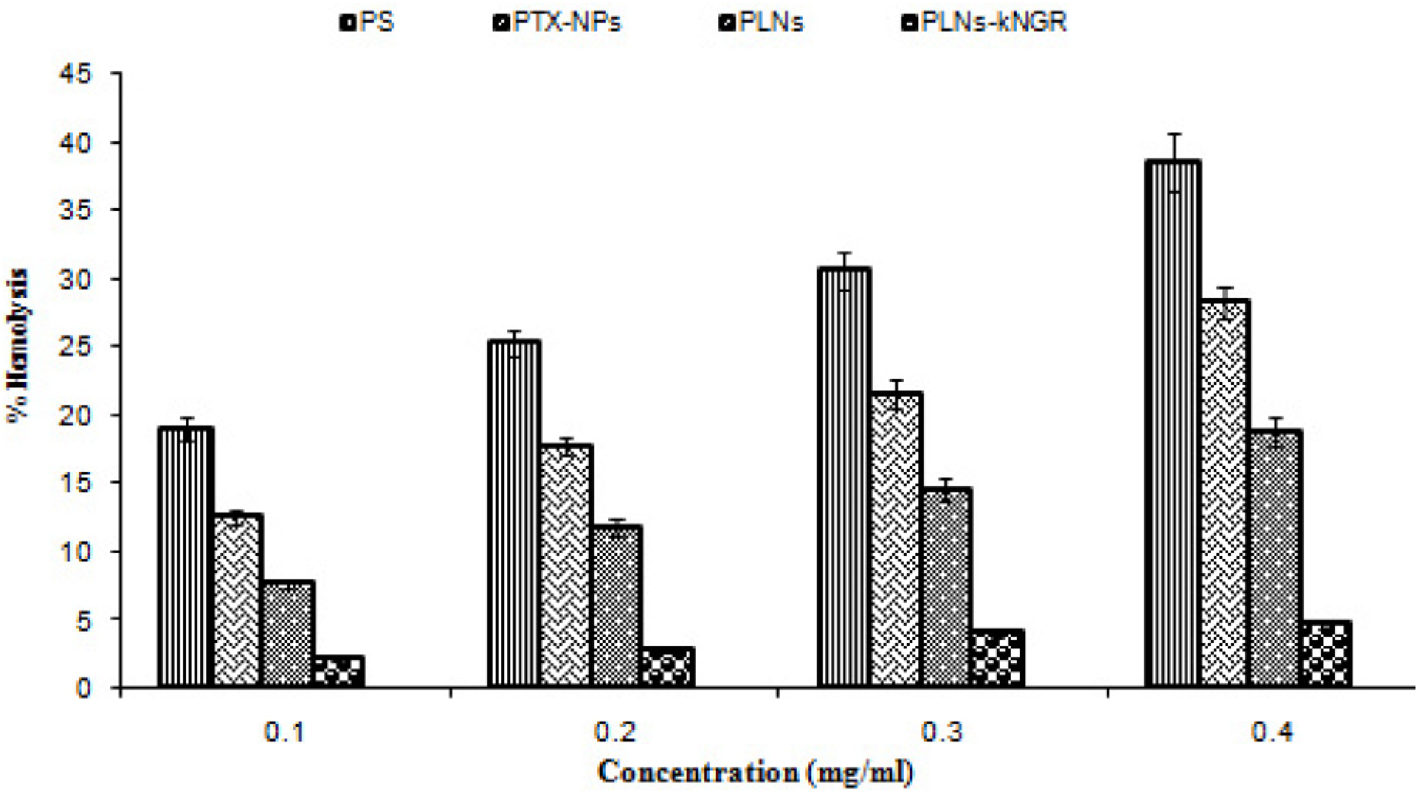

Figure S4: Hemolytic toxicity of free PTX and PTX loaded NPs formulations

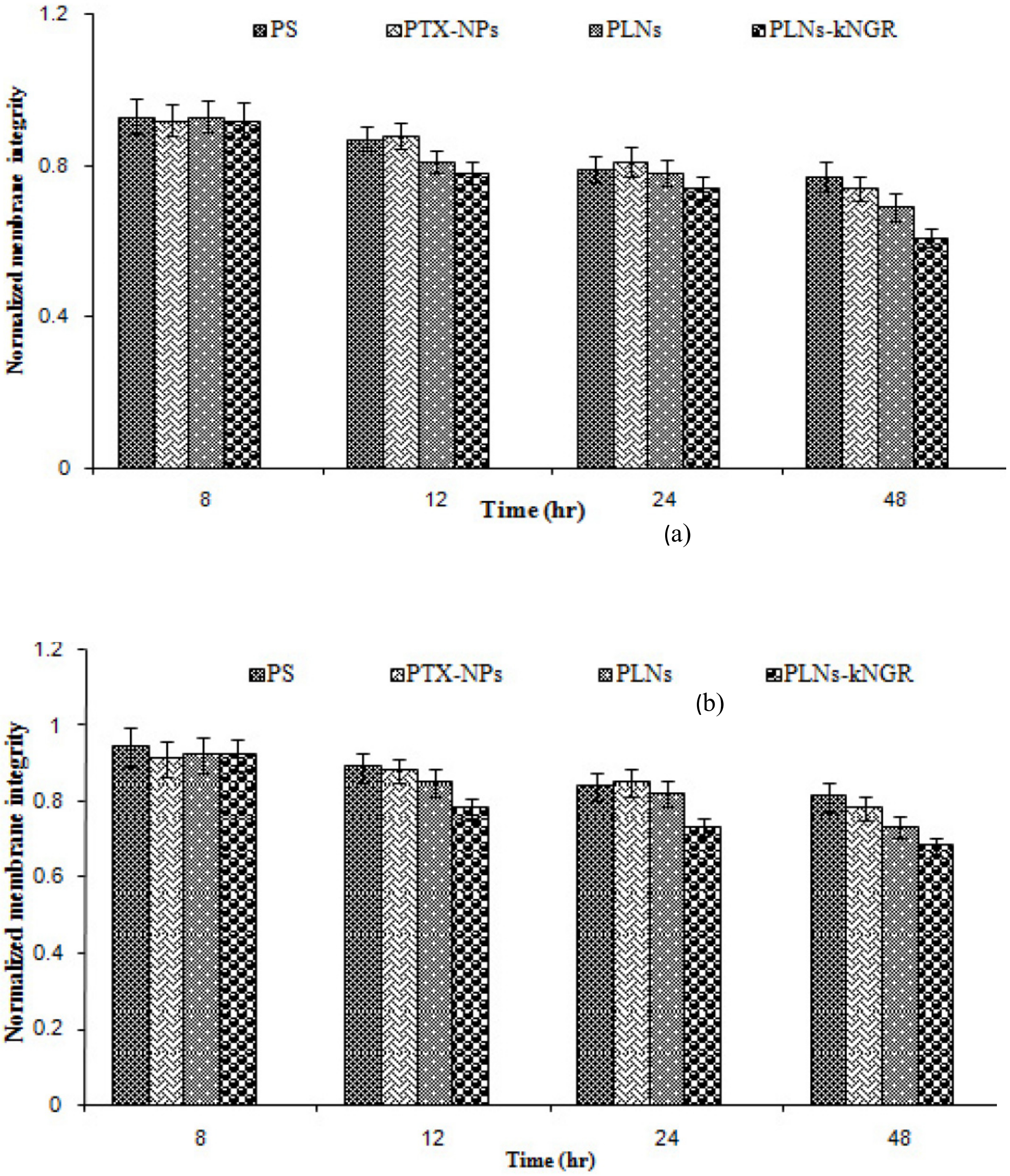

Figure S5: Results of trypan blue exclusion assay showing the effects of PTX solution (PS), PTX-NPs, PLNs and PLNs-kNGR on cell membrane integrity of (a) HT-1080 cells and (b) HUVEC cells. Cells wertreated for various time periods as 8, 12, 24 and 48 hr.

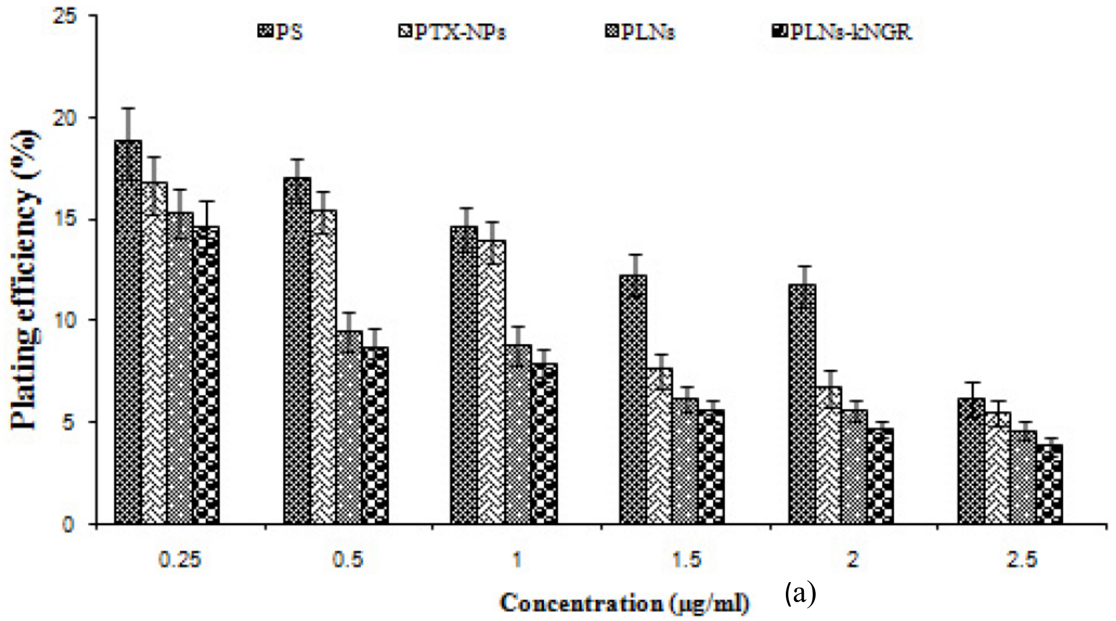

(a)

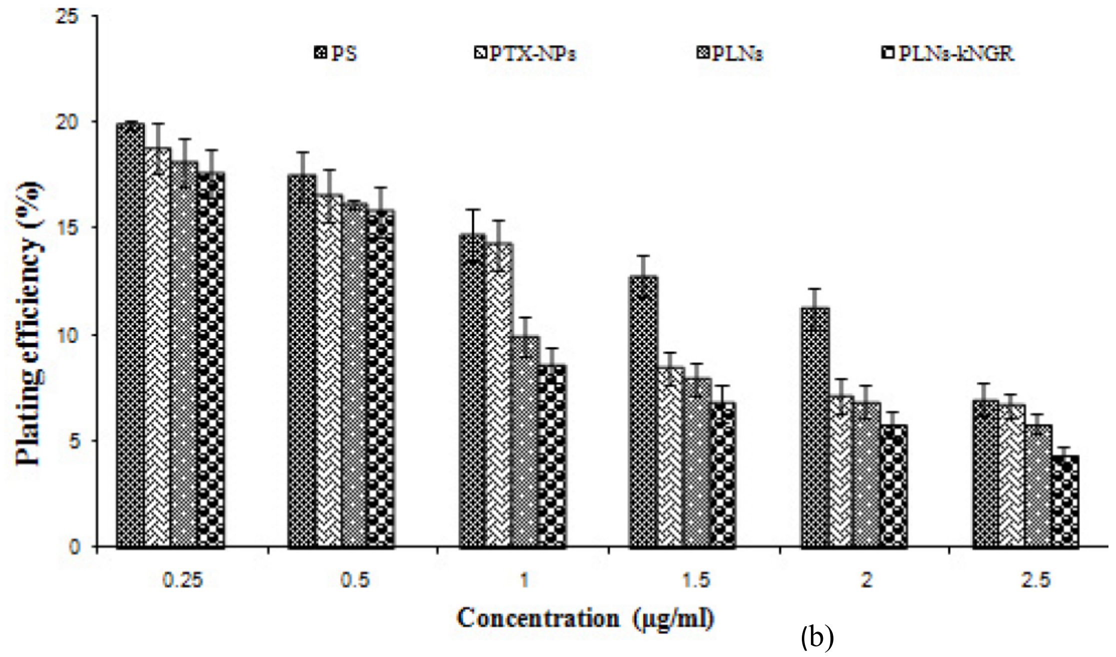

(b)

Figure S6: Clonogenic assay experiments for the toxicity of PTX based formulations in (a) HT-1080 cells (b) HUVEC cells lines. The plating efficiencies after 48 hr exposure to free drug solutions and various PTX based nanoparticulate systems are shown. Results are expressed as mean ±SD of the measurements obtained in three separate experiments (n = 6 in each experiment).

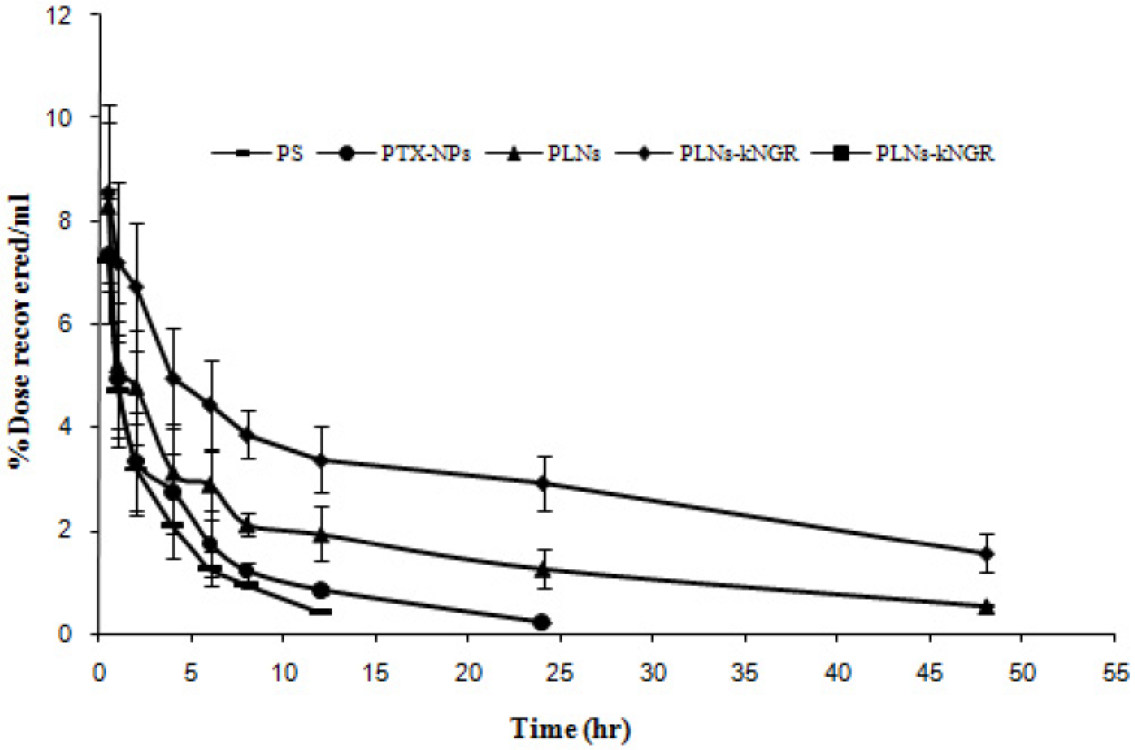

Figure S7: Plasma concentration-time curves of various PTX based formulations after i.v. administration to Balb/c mice at the same 10 mg/kg PTX dose.

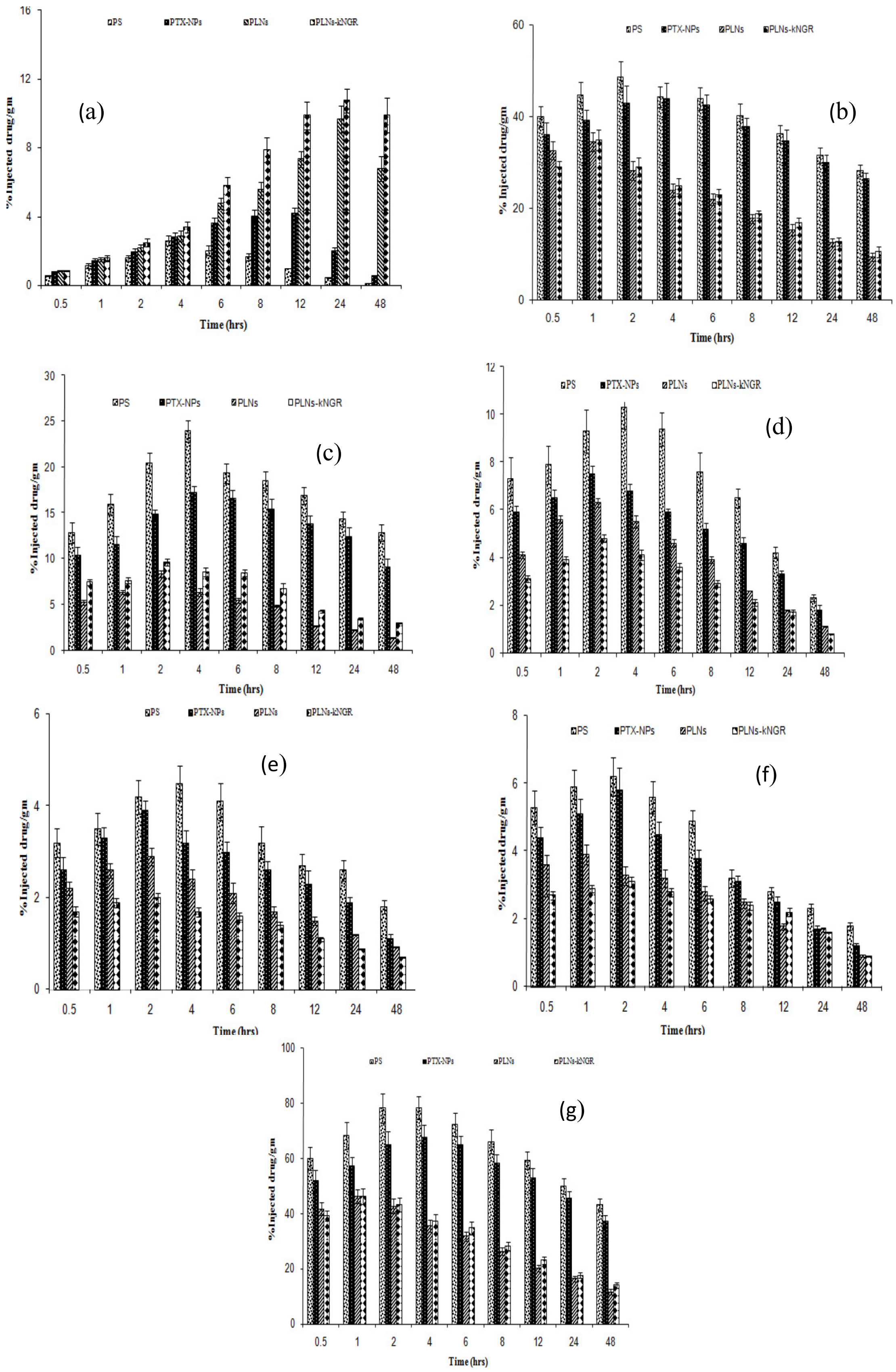

Figure S8: Biodistribution of PTX in tumor-induced Balb/c mice following intravenous treatment of several formulations at a dosage of 10 mg/kg. (a) tumor (b) liver (c) spleen (d) lung (e) heart (f) kidney (g)MPS
